# Mr. Thackeray, on Cold Water in Convulsions

**Published:** 1804-12-01

**Authors:** W. M. Thackeray

**Affiliations:** Chester


### To Dr. BATTY.

Sir,

In your Tenth Volume, p. 410, is inserted the Case of Miss V. The following is the result of that extraordinary case, which will probably prove acceptable to the Readers of the Medical Journal. I am, &c.

W. M. THACKERAY.

*Chester, October* 18, 1804.

---

On December 20, 1803, Miss M. V. felt a trifling oppression at her chest, accompanied with flatulency and pain.

On the 23d, She took her powder (pulv. gratiolæ ℈ß. pulv. zing. gr. v. 2dis horis per tres dies) which though operating violently, so far from relieving the oppression, seemed rather to increase it, and her rest at night was disturbed by a gnawing pain in her limbs, differing materially from the spasms, but which generally precedes them.

On

On the 25th, In addition to these symptoms, her hand was closed with the cramp, which was relieved by immersion in cold water.

On the 26th, She took a dose of calomel; every day the sensation of weight became more oppressive.

On the 28th, Her mouth, throat, and tongue were attacked by a severe spasm ; instant relief however was obtained from the cold water.

On January 6, The flatulency and pain continuing, she tried once more the gratiola powder ; it failed of affording its wonted relief. The oppression now became almost insupportable, and a sensation of violent pricking attended it, which was momentarily mitigated by either brandy or wine.

On the 8th, (as had been the case for many preceding evenings) About ten o'clock, violent pain and flatulency came on, when brandy and ætherial drops were ineffectually administered. About two in the morning she was apparently easy and composed for sleep, when suddenly she complained of an inability to move the right arm ; this want of sensation immediately extended itself to that whole side, and ere she could close her description of this partial suspension of feeling, the half articulated sound died on her lips, and every effort was made (ineffectually) to restore animation. The whole frame assumed and retained, whatever form or posture it was placed in; even those attitudes that, in health, are thought most painful long to continue in ; her breathing was free, and her pulse regular, though low, till a few minutes preceding her recovery, when it fluttered violently. A transient shivering now agitated her whole frame, and with a sudden and convulsive sigh she opened her eyes, as if awaking from a deep sleep, and complained of extreme weariness and languor. During the night she several times relapsed into this state of insensibility.

On the 9th, Though low and nervous, she sat at dinner with the family, from which she was called before the cloth was removed, to Mr. Cartwright. She complained to him of the oppression, &c. and declared her firm conviction that she was going to have a serious attack. While conversing with him, apparently free from pain, she suddenly ceased to speak, her unclosed eyes became fixed, and her person, retaining its erect position in the chair, seemed transformed into a statue, only that her pulse beat regularly, and her skin was of a comfortable warmth. Mr. C. ordered her in this state to be put under the shower-bath, which instantly instantly restored animation, but excruciating agony succeeded in her head, stomach, bowels, and limbs; occasional delirium, violent shivering, and during the paroxysm (which lasted nearly sixteen hours) relapsing several times into insensibility. She took eighty drops of tinct. op. and a musk draught, as well as a large quantity of brandy, but without any sensible benefit. The anguish in her stomach continuing, Mr. C. directed tobacco steeped in brandy to be applied as a poultice to her stomach. It seemed to afford a trifling alleviation of the spasm, but its subsequent effect was truly alarming; a death-like sickness supervened, accompanied by violent straining to vomit; she had cold and clammy perspiration, and her countenance was pale, livid, and distorted. She repeated, while delirious, long pieces of poetry with emphatic propriety, marking the passages that were peculiarly striking with an increased energy of enunciation, and occasionally observing on them with discriminating judgement.

The next day she appeared extremely debilitated, and at the usual hour at night, similar sufferings commenced, attended with a dejection of spirits, as unusual as distressing. The shower bath was had recourse to four times, and it never failed of affording relief, though sometimes the benefit was more apparent, sometimes more permanent, at other times merely transitory, the spasms returning a few minutes after emersion.

Wednesday night was ushered in by sufferings still more acute: for nearly three hours she was deprived of her speech; her head, stomach, and limbs were violently convulsed; then animation seemed wholly suspended, and her tortured frame, unequal to the struggle, bore every appearance of immediate dissolution. I now ordered her beverage to be iced, and large lumps of ice to be brought for trial; her teeth for some time had been fast closed, and by holding the ice a few seconds on her lips the muscles gradually relaxed. As if guided by instinct, she seized the ice, and rubbed her face, neck, and arms with it, signifying by gesture the ease it afforded. We then laid a piece on her stomach; the effect was as salutary as instantaneous, though, on its removal, the bowels again immediately contracted. We persevered in putting pieces of ice into her mouth, as the former ones dissolved, and finally had the satisfaction to find this experiment crowned with success; as it restored her speech, the deprivation of which had so greatly alarmed us.

The next night the paroxysm commenced about ten o'clock; o'clock, we then put her under the bath, and afterwards gave her 100 drops of tinct. op.; dreadful spasms succeeded in every part of the body, more especially the stomach and bowels, alienation of mind, suspended animation, in short, every symptom of this strange disorder. In the space of one hour we gave her 300 drops of tinct. op. with manifest advantage. The anguish seemed mitigated, and notwithstanding the delirium continued, it was unaccompanied with horror or distress. She held an imaginary conversation with a female friend, rallied her foibles with wit and spirit, exemplified her arguments with anecdotes, directing her voice, look, and action to vacuity. During four hours she took nearly 500 drops of tinct. opii, and was four times under the shower-bath.

On Friday evening her complaint returned at the usual hour, though the spasms were much less acute. Her stomach was greatly relieved by a strong assafœtida draught. She had long intervals of apparent cessation from pain, during which she not only repeated from various authors, as was her wonted practice, but attended to, and answered questions and observations that we put to her, with so much judgement, discrimination, and rationality, that, but for the peculiar turn of her countenance, unusual energy of her manner, and the total absorption of her mind in the subject that occupied it, (for though she would reply to any observation on that, with perfect precision, it was wholly impossible to turn the current of her ideas into another channel) we should scarcely have suspected in her any alienation of mind. Suddenly she ceased to speak, an universal rigidity pervaded her whole frame, and in about a quarter of an hour she uttered several sentences in an under tone of voice, like one who talks in his sleep, though articulately; she then began to be violently convulsed, and during the space of half an hour she seemed to suffer the extreme of torture. In this state we had her carried under the bath, which instantly restored her to ease and recollection. This night she took 400 drops of tinct. opii.

To the present period, Feb. 13, she has had no return of spasm, and is now able to walk two or three miles without the least fatigue. The shower-bath is continued every morning, and occasionally repeated in the evening.

P.S. I was in Shropshire last month, and saw my patient perfectly well. The young lady intends to persevere in the use of the shower-bath the whole of the approaching winter.

